# P-2165. Comprehensive Next-Generation Sequencing-Based Assay for Antimicrobial Resistance Gene Marker Identification in Urine

**DOI:** 10.1093/ofid/ofae631.2319

**Published:** 2025-01-29

**Authors:** Gabor Fidler, Mara Couto-Rodriguez, Xavier O Jirau Serrano, Heather L Wells, Sol Rey, Eduardo J Morfa Romero, Anna Plourde, Michael Augenbraun, Tiara Rivera, John C Papciak, Patrick F Combs, Caitlin Otto, Christopher E Mason, Niamh B O’Hara, Dorottya Nagy-Szakal, David C Danko

**Affiliations:** Biotia, Eger, Heves, Hungary; Biotia Inc., Brooklyn, New York; Biotia Inc., Brooklyn, New York; Biotia, Eger, Heves, Hungary; Biotia Inc., Brooklyn, New York; SUNY Downstate Health Sciences University, Brooklyn, New York; SUNY Downstate Medical Center, Brooklyn, New York; SUNY Downstate Medical University, Brooklyn, NY; Biotia, Eger, Heves, Hungary; Biotia Inc., Brooklyn, New York; Biotia, Eger, Heves, Hungary; Biotia Inc., Brooklyn, New York; Biotia Inc., Brooklyn, New York; Biotia Inc., Brooklyn, New York; Biotia Inc., Brooklyn, New York; Biotia, Eger, Heves, Hungary

## Abstract

**Background:**

Antimicrobial resistance (AMR) is a growing threat to human health contributing to 1.27 million global deaths annually. Due to the limited genetic repertoire necessary to confer bacterial resistance, new genetic variants regularly emerge. In the diagnosis of urinary tract infections (UTIs), culture and phenotypic resistance testing are considered the gold standard. However, due to their limitations (false negative culture, long turnaround time), inconclusive results can frequently lead to empirical treatment. Conversely, polymerase chain reaction (PCR)-based testing is fast, but primer design may not be able to keep up with rapid evolution and large numbers of potential genes. Next-generation sequencing (NGS) offers an effective path to rapid and comprehensive AMR diagnosis with “evolution-proof” assays.

**Methods:**

Culture positive clinical urine specimens (n=168) were processed with the clinical-grade BIOTIA-ID Urine NGS Assay including DNA extraction, library preparation, Illumina-NextSeq sequencing and BIOTIA-DX analysis to identify urogenital pathogens and AMR genes. Identified AMR genes were confirmed with qPCR. An additional cohort of culture negative (< 100k CFU/mL) urine specimens (n=201) were processed from patients with pyuria and UTI symptoms (Advarra Pro00038083, IRBNet 1950413-1).

**Results:**

We used NGS to identify seven AMR markers (TEM, SHV, sul1, KPC, CTX-M, vanA, mecA) directly in urine. NGS results were compared to PCR testing with corresponding accuracy between 96-100% for all tested genes. Of 369 specimens where a pathogen was identified 59.3% contained at least one AMR gene. Different AMR profiles could be distinguished based on the presence of different pathogens and AMR genes could generally be associated with specific pathogens. Beta-lactamases were the most prevalent type of AMR gene markers accounting for 55.3% of all identified genes.

**Conclusion:**

NGS is an effective tool to identify AMR markers directly from clinical urine specimens. It can identify AMR with high accuracy, can map AMR to a particular pathogen, and can be built into an existing NGS-based diagnostic assay. NGS is agnostic to the specific sequences of AMR markers; therefore, it can be used to detect and identify novel markers that arise through mutation.

**Disclosures:**

Gabor Fidler, PhD, Biotia: Employee of Biotia|Biotia: Stocks/Bonds (Private Company) Mara Couto-Rodriguez, MS, Biotia: Employee|Biotia: Stocks/Bonds (Private Company)|Biotia Inc: Employee|Biotia Inc: Stocks/Bonds (Private Company) Xavier O. Jirau Serrano, MS, Biotia Inc: Employee|Biotia Inc: Stocks/Bonds (Private Company) Heather L. Wells, MPH, PhD Candidate, Biotia, Inc.: Employee Sol Rey, BS, Biotia: Employee|Biotia: Stocks/Bonds (Private Company) Tiara Rivera, B.S., Biotia: Employee|Biotia: Stocks/Bonds (Private Company) John C. Papciak, BS, Biotia: Employee|Biotia: Stocks/Bonds (Private Company) Patrick F. Combs, PhD, Biotia: Employee Christopher E. Mason, PhD, Biotia: Board Member|Biotia: Honoraria|Biotia: Ownership Interest|Biotia: Stocks/Bonds (Private Company) Niamh B. O'Hara, PhD, Biotia: Board Member|Biotia: Two patents related to genomic sequence analysis of microbial species|Biotia: Ownership Interest|Biotia: Stocks/Bonds (Private Company) Dorottya Nagy-Szakal, MD PhD, Biotia Inc: Employee|Biotia Inc: Stocks/Bonds (Private Company) David C. Danko, Ph.D., Biotia Inc: Employee|Biotia Inc: Stocks/Bonds (Private Company)

